# Concentrations of ^137^Cs and ^40^K radionuclides and some heavy metals in soil samples from the eastern part of the Main Ridge of the Flysch Carpathians

**DOI:** 10.1007/s10967-013-2890-3

**Published:** 2014-01-07

**Authors:** Barbara Kubica, Katarzyna Szarlowicz, Marcin Stobinski, Stefan Skiba, Witold Reczynski, Janusz Gołas

**Affiliations:** 1 Faculty of Energy and Fuels, AGH University of Science and Technology, al. A. Mickiewicza 30, 30-059 Kraków, Poland; 2 Faculty of Biology and Earth Sciences, Jagiellonian University, ul. Gronostajowa 7, 30-387 Kraków, Poland; 3 Faculty of Materials Science and Ceramics, AGH University of Science and Technology, al. A. Mickiewicza 30, 30-059 Kraków, Poland

**Keywords:** ^137^Cs, ^40^K, Soil, Heavy metals, Gamma spectrometry

## Abstract

The aim of the study is to present the results of determination of radioactivity of artificial ^137^Cs and natural ^40^K and certain heavy metals in soil samples collected from the eastern part of the Main Ridge of Carpathians, including the Beskid Niski Mts and the Bieszczady Mts. The evaluation of level of radionuclides was based on the bulk density analysis of the soil. A valuable finding of the study was a good linear correlation between the level of ^137^Cs concentration and bulk density of the soil as well as an inverse correlation between radioactivity of natural ^40^K and tested soil density. This might indicate though a high competitiveness of these elements between each other. Moreover, a good correlation between the concentrations of artificial element ^137^Cs and Pb has been also observed in soil samples collected from the Beskid Niski Mts. In most cases, the level of artificial ^137^Cs was lower comparing to an average ^137^Cs concentration established for soils in Poland.

## Introduction

The natural environment is exposed to various chemical contaminants, including the radioactive elements. Some of these radioactive elements are long-lived naturally occurring radionuclides. These radionuclides have always been present in the Earth’s crust and atmosphere. There are approximately 60 natural radionuclides and one of the most abundant elements in the Earth’s crust is ^40^K that remains to this point in time. Another source of radioactivity has a cosmogenic origin and it is the result of interaction between certain gases in the Earth’s atmosphere and cosmic rays [1]. Besides such sources of naturally occurring radiation exposure, the natural environment may be subjected to radioactive contamination caused by human activity. The main sources of anthropogenic radioactive material were (and still are) nuclear weapons tests conducted in the atmosphere, the accidents of nuclear power plants, and either the processing or storage of nuclear fuel and large amounts of nuclear waste [2–4]. An example of the world’s worst civilian nuclear disaster was spreading into the atmosphere of Central and Eastern Europe a massive amount of radiocaeasium due to the explosion and fire at the Chernobyl power plant in April 1986.

The current state of knowledge concerning radioactivity on the whole area of Polish mountain’s soils is still not comprehensive. Though, to address this knowledge gap, a radioactivity study was carried out from 2011 to 2012 by the Department of Coal Chemistry and Environmental Sciences. The experimental area covered the eastern regions of the Main Ridge of the Flysch Carpathians Belt (the areas including the Beskid Niski Mts and the Połoniny part of the so-called Tarnica Mt in the Bieszczady Mts). The survey was conducted with the sampling and analysis of 27 soil samples in terms of spatial distribution of ^137^Cs and natural ^40^K and certain heavy metals including Cu, Zn, Cd and Pb. Data presented herein provide an update of a national evaluation regarding radioactivity in soils in the southern part of Poland since 2000, which included monitoring carried out in the Central Carpathians (the Tatra Mts) [5–8] and part of the East Carpathians area (the Chornohora Mts) [9].

## Experimental

### Sampling and methods

To collect the soil samples, 27 sampling locations were selected. Sampling was started in June 2011. The measurements of the gamma emitting radionuclide activity as well as the concentrations of certain heavy metals were performed on soil samples collected from the surface horizons of soil above 500 m ASL. The experimental area includes the Połoniny Caryńska and Wetlińska Mts, “Gniazdo Tarnicy” (Rozsypaniec, Halicz, Krzemień, Tarnica, Tarniczka Mt) in the Bieszczady Mts and the Beskid Niski Mts (specifically, Jaworzyna Krynicka, Spalone and Wołowiec close to Zawoja stream). Detailed positional data of the selected sampling points were established by a Garmin GPS Map 76CS satellite navigation system.

### Sampling method and preparation of the samples

Soil samples were collected by the use of a cylidrical sampler that provides “soil cores” (10 cm height, 10 cm diameter). Those cores were cut into three segments that represented different soil layers starting from the soil surface: 0–3, 4–6, 7–10 cm (samples: a, b and c, respectively). That procedure allowed collection of 3 samples from each sampling point. The samples were dried at 105 °C to constant weight (for about 3 days), then the bulk density was determined and the samples were sieved (mesh diameter = 2 mm). The samples were prepared and stored prior to measurements in polyethylene containers.

## Analytical procedure

### Gamma spectrometric analysis

Measurements of individual gamma radionuclides performed using a gamma spectrometer equipped with HPGe detector (20 % relative efficiency) with the resolution of 1.9 keV at 662 keV (gamma spectrometer Canberra Packard, type GC2020, P-type Coaxial Germanium Detector, USA). The spectrometer was calibrated with the appropriate standards from the International Atomic Energy Agency in Vienna (IAEA-154,375 and 447). The analysis of all spectra was performed using the Ortec Maestro and Canberra Genie-2000 software. The single sample measurement time was 259,200 s (72 h). Some metrological parameters of the applied techniques were evaluated. Considering ^137^Cs concentration, MDA (minimum detectable activity) changes with weight of measured soils were determined. It was 2.3 Bq/kg (~70 g soil) and 7.1 Bq/kg (~11 g soil). The MDA for ^40^K determination varied between 50 (~70 g soil) and 161 Bq/kg (~11 g soil). The accuracy of the developed analytical procedure was checked by the analysis of the certified reference soil material. Satisfactory agreement with the certified values of concentrations of the radionuclides was achieved. Better agreement was achieved for the samples with higher content of measured radionuclides, this resulted in a higher number of counts per second in net area peak (1.5 % for the IAEA-375). Uncertainties of the values presented in Tables 1 and 2 in majority were around 5 % and weren’t higher than 10 % (k = 2).

**Table 1:** Detailed location of the sampling points given for the Beskid Niski Mts and activities of ^137^Cs and ^40^K presented in Bq per mass units or per surface units together with soil samples bulk density

No	Location	Activity, dry mass				Altitude m ASL	Bulk density g/cm
		^137^Cs Bq/kg	^137^Cs Bq/m^2^	^40^K Bq/kg	^40^K Bq/m^2^		
0	Jaworzyna Krynicka 49°25′15″ N 20°53′12″E	52	440	242	2,069	1,049	0.49
1	Near Waholowski Wierch 49°24′38″ N 22°02′30″E	25	364	537	9,160	605	0.97
2	On the way to Tokarnia 49°25′41″ N 22°02′29″E	63	843	702	10,035	762	0.82
3	Near Tokarnia 49°25′16″ N 22°03′13″E	16	202	672	8,944	518	0.76
4	On the way to Wilcze Budy 1 49°26′30″N 22°00′37″E	111	1,375	623	7,727	723	0.73
5	On the way to Wilcze Budy 2 49°26′53″N 21°59′43″E	40	709	612	6,534	719	0.72
6	On the way to Puławy Górne 49°28′45″N 21°56′25″E	48	303	646	11,336	720	1.0
7	Before intersection the trail Red and green on the way to Skibice 49°28′32″N 21°56′21″E	227	2,972	517	3,252	743	0.63
8	On the way to Skibice and the Kozie Żebro Mt (about 45 min.) 49°28′27″N 21°56′36″E	1,127	8,463	253	1,901	758	0.43
9	The entrance to Kozie Żebro 49°27′52″N 21°12′40″E	322	4,364	78	1,030	637	0.76
10	On the way to Rotunda from Regietów 49°28′16″N 21°13′36″E	514	7,647	114	1,548	684	0.38
11	On the way to PopoweWierchy 49°30′12″N 21°15′51″E	165	1,378	316	4,707	554	0.48
12	On the way to Wołowiec near the stream Zawoja 49°30′23″N 21°19′11″E	245	2,039	618	2,275	558	0.48

**Table 2:** Detailed location of sampling points given for the Bieszczady Mts and activities of ^137^Cs and ^40^K presented in Bq per mass units or per surface units together with soil samples bulk density

No	Location	^137^Cs Bq/kg	^137^Cs Bq/m^2^	^40^K Bq/kg	^40^K Bq/m^2^	Altitude m ASL	Bulk density g/cm^3^
13	Smerek Mt 49°11′07″N 22°28′45″E	250	2,312	499	4,605	1,189	0.61
14	Połonina Wetlińska 3 49°09′26″N 22°33′05″E	201	1,674	317	2,633	1,218	0.49
15	Połonina Wetlińska 2 49°09′58″N 22°31′41″E	143	1,385	507	4,902	1,206	0.67
16	Połonina Wetlińska 1 49°10′06″N 22°31′06″E	218	873	585	2,348	1,182	0.48
17	Połonina Caryńska 2 49°08′18″N 22°36′09″E	171	1,216	389	277	1,294	0.52
18	Połonina Caryńska 1 49°08′07″N 22°36′26″E	230	1,338	441	2,557	1,247	0.41
19	TarnicaMt 49°04′37″N 22°43′30″E	163	1,432	50	5,522	1,324	0.65
20	Tarniczka Mt 49°04′46″N 22°43′24″E	180	1,885	844	9,085	1,293	0.63
21	Krzemień Mt 49°05′07″N 22°44′22″E	500	1,865	220	844	1,314	0.23
22	Krzemień Mt 49°04′52″N 22°44′51″E	374	1,107	266	825	1,251	0.24
23	Halicz Mt 49°04′43″N 22°45′59″E	609	3,505	293	1,685	1,250	0.41
24	Halicz Mt 49°04′21″N 22°46′08″E	198	2,095	916	9,685	1,323	0.65
25	Rozsypaniec Mt. 49°03′45″N 22°46′12″E	356	1,594	196	8,989	1,261	0.34
26	Rozsypaniec Mt 49°03′22″N 22°46′06″E	236	1,596	221	1,496	1,204	0.52

The radioactivity level of artificial ^137^Cs and natural ^40^K was shown in two modes:

1. the radioactivity of ^137^Cs in the upper core part (up to 10 cm) [Bq/m^2^];
2. the concentration of ^137^Cs per mass unit [Bq/kg] in each of tested soil layers (samples a, b, c).

## Atomic absorption spectrometry (AAS) analysis

Sieved and homogenized soil samples were wet digested in the microwave system (Anton Paar Multiwave 3000, Switzerland) with concentrated HNO_3_ and HClO_4_ (Merck, Germany). After digestion, the samples were transferred into quartz crucibles and the excess of reagents was evaporated on a hot plate and the residue was transferred quantitatively to volumetric flasks. Quadruple distilled water was used for glassware preparation and sample dilution. Quantitative determination of Zn and Cu was performed using flame technique at standard conditions (AAS spectrometer Perkin Elmer, Model 3110, USA) and concentrations of Cd and Pb were determined using electrothermal technique (AAS spectrometer with Zeeman background correction, Perkin Elmer 4100 ZL, Germany). Graphite furnace parameters were optimized using the Method development program to obtain high sensitivity and precision of measurements for each of the elements determined by means of ET AAS technique. Uncertainties of the elements quantitative determinations (RSD data presented in Table 2) were not higher than Cu 5.4 %, Zn 2.6 %, Cd 5.6 %, Pb 4.7 %. The accuracy of the analytical procedure was estimated by the use of the certified reference material (IRMM BCR-280, Lake Sediment). Satisfactory accuracy and precision was achieved.

## Results and discussion

The surface horizons of soil consist of organic matter in various stages of humification, lying directly on the weathered rocks flysch substrate initial soils -Lithic Leptosols or as the level of accumulation in Rankers –Umbric Leptosols, Cambic Leptosols (Skeletic) and Dystric Cambisols [10]. In this survey, the material belonging to initial and poorly formed types of soil was analyzed. It forms the specific structure of the soil cover occurring above the 500 m ASL. The soils are rocky outcrops of initial soils—Lithic Leptosols and Rankers Cambic Leptosols. On the Pleistocene regolith—initial soils were formed Hyperskeletic Regosols. Characteristic properties of all mountain soils including the soils of three searched areas of the Carpathians (Tatra Mts and Charnohora Mts) are as follows: decreasing pace of organic matter decomposition linked to the increase of altitude above the sea level and the presence of ectohumus horizons in the depths. The organic matter of those horizons shows similar chemical properties regardless the parent rock. Thus, there are similar forms of ectohumus both in the initial soils (Lithic Leptosols), rankers (Umbric Leptosols), rendzinas (Rendzic Leptosols)and in Podzols [11]. Below this level, there is little stale bedrock (typically sandstones) or weathering hyperskeletic regolith of flysch rocks.

**Fig. 1:**
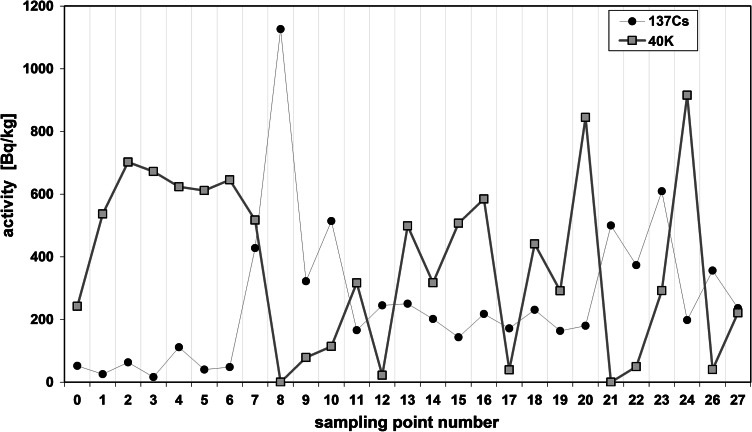
Spatial distribution of radioactivity [Bq/kg] of gamma radionuclides (^137^Cs and ^40^K) in the 27 samples of mountain soil collected from the Beskid Niski Mts and the Bieszczady Mts

**Fig. 2:**
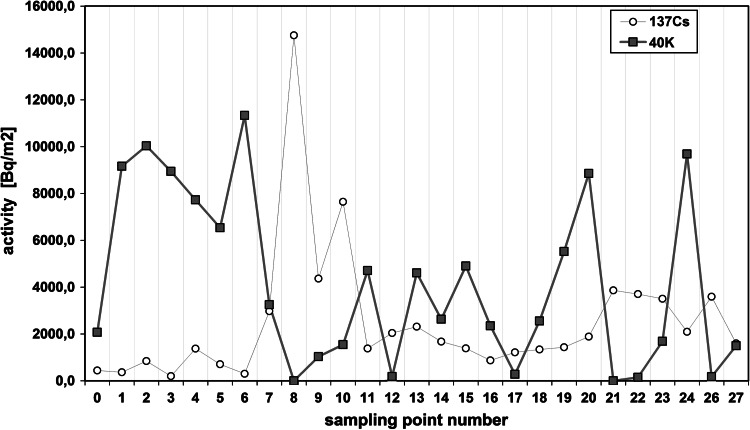
Spatial distribution of radioactivity presented in Bq per surface [m^2^] of gamma radionuclides (^137^Cs and ^40^K) in the 27 samples of mountain soil collected from the Beskid Niski Mts and the Bieszczady Mts

**Fig. 3:**
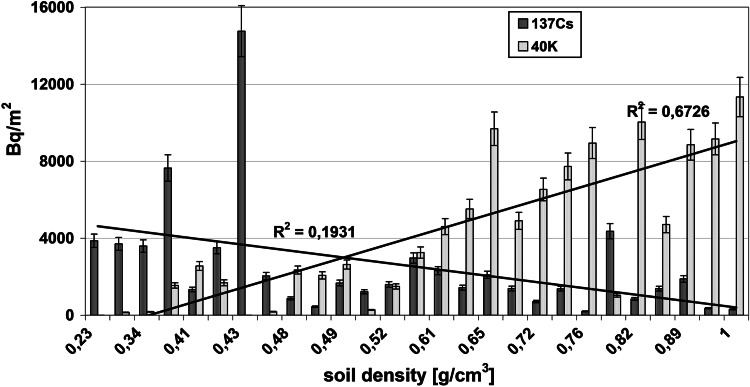
Correlation between the gamma radionuclides activity (^137^Cs and ^40^K) and the density of the tested soil samples

**Fig. 4:**
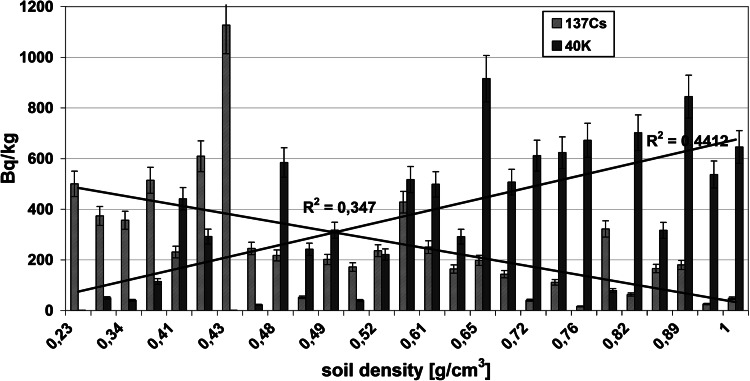
Correlation between the gamma radionuclides activity (^137^Cs and ^40^K) measured in the surface layer “a” (0–3 cm) of the collected soil cores and the density of the tested soils

**Fig. 5:**
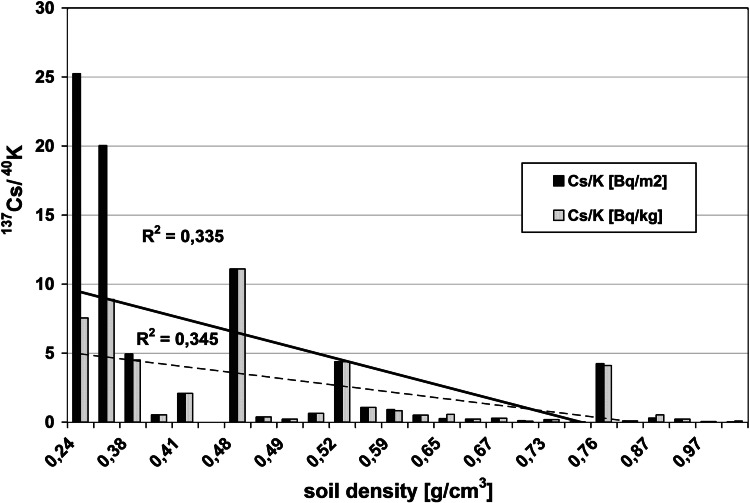
Radioceasium-137/potassium-40 activity ratio presented as a function of the density of tested soil samples

The radioactivity of artificial ^137^Cs and natural ^40^K in the soils collected from the Main Ridge of the Flysch Carpathians, specifically from region of the Beskid Niski Mts and from the region of the Bieszczady Mts is given in Tables 1 and 2, respectively. The data obtained from selected soil covers (the Beskid Niski Mts and the Bieszczady Mts area) showed high variation. The ^137^Cs concentration in the Beskid Niski Mts area ranged between 16 and 1,137 Bq/kg in the first layer (a-layer) of soil cores and from 843 to 8,463 Bq/m^2^ for the entire 10 cm depth soil core (Tab 1). The concentration of ^137^Cs in the Bieszczady Mts varied from 143 to 609 Bq/kg (for the layer “a”) and from 1,385 to 3,505 Bq/m^2^ for the sum of the layers a, b and c (Table 1). It is noticeable, that the ^137^Cs radioactivity level was relatively low (about 107 Bq/kg) in the Beskid Niski Mts with exception of three sampling points located near Kozie Żebro Mt (points 8–10) (Table 1; Fig. 1). Fluctuations of the radionuclides concentration in the soils could strongly depend on the meteorological conditions. Thus, the elevated activity of ^137^Cs around Kozie Żebro Mt might have contributions from increased precipitation that occurred in May and June 1986 year (approximately 119–167 dm^3^/m^2^). In April that year, the rainfalls observed in the Beskid Niski Mts as well as in the Bieszczady Mts (apart from a few of small regions—Kozie Żebro) were rather small (24 dm^3^/m^2^) [12]. As mentioned above, the concentration of ^137^Cs in the western part of the Beskid Niski was low, however, the radioactivity rapidly increased close to Skibice 1,127 Bq/kg, (8,463 Bq/m^2^, sampling point no 8) and again was slightly reduced to approximately 165 Bq/kg (1,378 Bq/m^2^, sampling point no 11). This trend is also observed in the Bieszczady Mts, both on Połonina Wetlińska and Caryńskie (sampling points 14–18) with noticeable elevation of ^137^Cs occurred in the so-called “Socket Tarnica” (sampling point 19). All sampling sites with high level of ^40^K showed low concentration of ^137^Cs (Figs. 1, 2). These two isotopes belong to the same group of alkali metals, have similar chemical properties and are strong reducing agents. That is an explanation for competitive-sorption behavior of these two elements in the soil. The competitiveness between ^40^K and ^137^Cs in mountain soils has been already demonstrated in the previous studies [8–11, 13]. Also, for all sampling sites, the correlations between the ^137^Cs level, altitude and soil bulk density were found. It is well documented, that mountain soil properties are altered with the altitude, particularly the features of upper soil layers (O horizon, first 10 cm). In the surface soil layers, the level of organic material increases with the altitude (included the subalpine zone). It is noticeable, that not only the quantity of organic mass changes but also its decomposition stages and the content of humic and fulvic acids (the ratio of humic acids in humus organic matter) [14]. The humus modification implies the perturbations on soil bulk density, contributes to the small immobilization of radiocaesium in organic part of soils and hence to its higher availability to plants [15]. In this context, the relation between soil bulk density and ^137^Cs concentration might reflect the interdependence between the altitude and ^137^Cs concentration. The radioactivity obtained for both gamma emitters was also presented in relation to the volume density of the soils (Figs. 3, 4). The concentration of ^137^Cs underwent massive fluctuations in sampled soils however, we found a good linear correlation between ^137^Cs radioactivity expressed in mass units in the first layer (a) of soil core, and the soil density (Fig. 4). This correlation was less clear when comparing the radioactivity of this radionuclide expressed in units of surface (Fig. 3) and calculated for the entire 10 cm layer of soil a, b and c. There was significantly better correlation between the activity of ^40^K and the soil density observed in the Beskid Niski Mts and the Bieszczady Mts area. It is also noticeable, that the radioactivity of ^137^Cs and ^40^K per area unit corresponded well to the concentrations of these radionuclides expressed per mass units. Obtained results are in line with the data from similar studies carried out on the other mountain areas (the Tatra Mts and the Charnohora Mts). Our findings also confirmed the hypothesis of the radioactive caesium sorption in the organic soil layers. With increase of the soil density, the concentration of ^137^Cs decreased and in the same time, the amount of natural ^40^K increased what was shown in Figs. 1 and 2. For all sampling points, the ratio of ^137^Cs activity to the potassium (both for the first layer “a” and for the whole profile) presented as the function of the soil density was calculated (Fig. 5). Given results might also indicate that in some cases ^40^K was present not only in the mineral, but also in organic layer of the tested soils.

### Heavy metals level in soils of the Beskid Niski Mts area

**Fig. 6:**
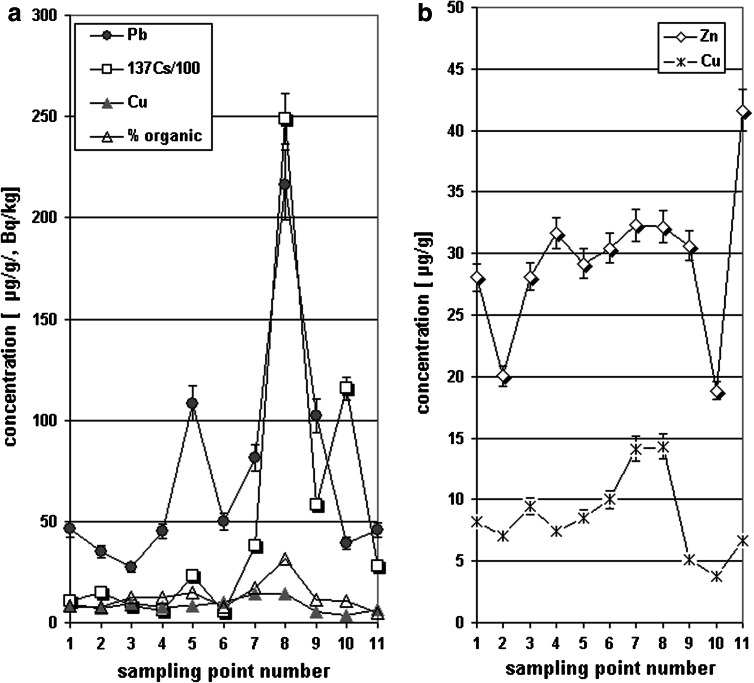
Spatial distribution of the level of anthropogenic (**a**) and natural (**b**) elements in the soils samples collected from 11 different localizations in the Beskid Niski Mts

The results of quantitative analysis of the selected metals (Cu, Zn, Cd and Pb) is presented in Table 2; Fig. 6a, b. The given values are the mean of 4 repetitions of the element concentration measurements. Copper concentrations were in the range from 3.77 to 14.31 [μg/g], zinc in the range 17.50 to 41.63 [μg/g]. The level of elements considered as toxic—Cd and Pb were in the ranges 0.18–1.10 [μg/g] and 27.27–216.10 [μg/g], respectively. Pb concentration in the soils collected from three locations (sampling point no 4,8 and 9) was substantially higher comparing to all others (see Table 2; Fig. 6a), probably due to higher amount of precipitation observed in these sampling areas.

In Fig. 6a changes of the elements (being the constituents of inorganic material) concentrations in relation to the soil sampling point number are presented. The sampling point elevation ASL of the sampling points differed only a little in the considered geographical region (see Tab. 1).

The concentration fluctuations observed for Zn and Cu in tested soil samples were similar for both elements (Table 2; Fig. 6b). It indicates that these elements predominantly originate from natural sources (mother rock erosion) and that mineral composition of Flysch Carpathian Belt in sampling area is more or less homogenous. The changes of the anthropogenic origin elements i.e. Pb and ^137^Cs (presented in μg/g for Pb and activity Bq/kg units for ^137^Cs) with percentage of organic matter in tested soils were showed in Fig. 6a. The fluctuations in the concentration of Pb and^137^Cs were related to the organic matter content and can indicate Pb and Cs immobilization in organic matter. The findings were clearly visible in the sampling points no 7,8 and 9—the level of elements increased with the amount of organic matter (Table 2; Fig. 6a).

## Conclusions

1. The content of ^137^Cs, ^40^K and certain heavy metals in soils collected from the eastern Carpathians areas was found to be greatly variable: The Beskid Niski Mts area—from 25 Bq/kg (364 Bq/m^2^) for the Waholowski Peak (605 m,sampling point 1) to 1,127 Bq/kg (8,463 Bq/m^2^) for the Kozie Żebro Mts (758 m, sampling point 8); The Bieszczady Mts area—from 143 Bq/kg (1,385 Bq/m^2^) for the Połonina Wetlińska 2 (1,206 m ASL, sampling point 15) to 609 Bq/kg (3,505 Bq/m^2^) for the Halicz Mt (1,250 m ASL, sampling point 23).
2. Radioactive cesium activity in the mountain soils showed the tendency to decrease with increasing density of the soil due to high amount of the organic matter. The humus, as the main component of the sorption complex, was responsible for the immobilization of ^137^Cs ions.
3. Our results confirmed strong competitive-sorption behavior of ^137^Cs and ^40^K in the soil sorption complex.
